# Predictors of Prolonged TB Treatment in a Dutch Outpatient Setting

**DOI:** 10.1371/journal.pone.0166030

**Published:** 2016-11-10

**Authors:** Natasha van’t Boveneind-Vrubleuskaya, Alper Daskapan, Jos G. W. Kosterink, Tjip S. van der Werf, Susan van den Hof, Jan-Willem C. Alffenaar

**Affiliations:** 1 Department of Public Health TB Control, Metropolitan Public Health Service, The Hague, The Netherlands; 2 University of Groningen, University Medical Center Groningen, Department of Clinical Pharmacy and Pharmacology, Groningen, The Netherlands; 3 KNCV Tuberculosis Foundation, The Hague, The Netherlands; 4 Amsterdam Institute for Global Health and Development, Amsterdam, The Netherlands; 5 University of Groningen, University Medical Center Groningen, Department of Pulmonary Diseases and Tuberculosis, Groningen, The Netherlands; Hebrew University, ISRAEL

## Abstract

**Introduction:**

Standard treatment duration for drug-susceptible tuberculosis (TB) treatment is 6 months. Treatment duration is often extended—and for various different reasons. The aim of this study was to determine the prevalence and to assess risk factors associated with extended TB treatment.

**Methods:**

A cross-sectional study was conducted. Data including demographic, clinical, radiological and microbiological information from the Netherlands TB Register (NTR) of 90 patients with smear and culture positive pulmonary TB of the region Haaglanden, The Netherlands, was eligible for analysis.

**Results:**

Treatment was extended to ≥ 200 days by 46 (51%) patients. Extended TB treatment was associated with a higher frequency of symptoms, presumed to be due to adverse drug reactions (ADR; OR 2.39 95% CI: 1.01–5.69), drug-induced liver injury (DILI) (OR: 13.51; 95% CI: 1.66–109.82) and longer than 2 month smear and culture conversion rate (OR: 11.00; 95% CI: 1.24–97.96 and OR: 8.56; 95% CI: 1.53–47.96). In the multivariable logistic analysis, development of DILI emerged as the single statistically strong risk factor necessitating extension of TB treatment.

**Conclusion:**

This finding will need further confirmation in a prospective study, exploring the possible mutual role of pharmacokinetic and pharmacogenetic determinants of DILI among TB patients.

## Introduction

According the WHO guidelines the aims of TB treatment are to cure the patient, to prevent death from active TB; to prevent relapse of TB; to reduce transmission of TB and to prevent the development and transmission of drug resistance TB strains [1]. In general, TB treatment outcome is usually successful [2,3] but some factors may have a negative impact on the course of the TB treatment: extent of disease, the drug of *Mycobacterium tuberculosis* (Mtb), previous TB treatment, comorbidity, use of more than 6 co-prescribed drugs and adverse drug reactions [4–6]. The application of Directly Observed Treatment (DOT) and improvement of TB treatment outcomes is still discussed as results differ among studies [7–9]. Several studies showed that suboptimal treatment resulted in a prolonged time to culture conversion and therefore increased duration of treatment [10,11,12]. The underlying problems may be the inter- and intra-individual variation in drug concentration; and the occurrence of ADR [13,14]. Early detection of risk factors predisposing for lack of treatment response or ADR is therefore priority during TB treatment.

Based on WHO and national Dutch guidelines, drug-susceptible TB should be treated for 6 months: first a 2-month intensive phase with 4 drugs and then a 4-month continuation phase with two drugs [1,15,16]. However in some situations this period should be extended. Prolonged treatment is indicated, for example, for patients with drug-susceptible pulmonary TB who have cavitation on the initial chest X-ray and still have positive sputum culture results at the end of the intensive phase [17]. Extension of TB treatment may improve treatment outcomes but it is presently unclear why these delayed therapeutic responses occur. If delayed response to treatment, or the occurrence of ADR, is driven by anti TB drug exposure, therapeutic drug monitoring (TDM) could help to detect such under- or overexposure [18]. TDM is not mentioned in WHO treatment guidelines and therefore not part of standard treatment. TDM has been used in in selected cases mainly in TB referral Centers [19]. TDM might impact on duration of therapy, assuming that identification of dose-related overexposure resulting in ADR requiring interruption of therapy, as well as underexposure resulting in delayed sputum culture conversion result in extended treatment duration. Before TDM is introduced in routine care it would help to identify the prevalence of extended treatment and risk factors associated with it.

The objective of this cross-sectional study was therefore to identify the prevalence of and risk factors contributing to an extension of TB treatment for drug susceptible TB in a Dutch outpatient setting.

## Materials and Methods

### Study subjects

All patients from the region Haaglanden, The Netherlands, diagnosed with smear and culture positive pulmonary drug-sensitive TB in 2010–2013 and registered as having completed TB treatment, were eligible for inclusion in this study. Patients were treated by specialists of general hospital in our region or by specialist from TB department of the GGD (Municipal Public Health Service). Patients were excluded from the study, if (a) the surveillance data from the national tuberculosis registry could not be completed by in-patient medical records; (b) patient died during the treatment, or (c) were lost to follow-up.

### Study design and methods

Anonymized surveillance data of all notified culture positive TB cases registered in region Haaglanden, between January 2010 and December 2013, were received from the NTR and supplemented with data obtained from patient’s medical records by the first author (NB). NB was attending physician at GGD Haaglanden and performed the combination of the data. Subsequently, identifiers were removed and the database was locked. Analyses were performed with anonymized data. Co-authors had only access to anonymized data.

Patients were divided into two groups based on duration of treatment. A ‘TB patient with prolonged treatment’ was defined as patient with smear- and culture- positive pulmonary drug-sensitive TB, treated ≥ 200 days. ‘Patients with standard TB treatment duration’ were defined as patients with smear- and culture positive pulmonary TB, treated ≤ 199 days. The cut-off of 200 days was chosen based on summation of 6 months of standard TB treatment plus 2 weeks. The addition of 2 weeks was chosen, because at least 2 weeks interruption of TB treatment in the intensive phase for any reason leads to reintroduction of intensive phase treatment and thereby to extension of total TB treatment duration.

We conducted a cross-sectional study with demographic, TB diagnosis and TB treatment patient characteristics. The following individual-level variables were studied: age, sex, weight, race, alcohol and drug addiction, disease site, presence of cavitary TB, history of TB treatment, time to sputum smear and culture conversion, creatinine level, implementation of DOT, suspicion of ADR based on symptoms, co-morbidity, DILI, hospitalization in TB referral center and TB treatment by TB Municipal Public Health Service or specialist from hospital.

Suspected ADR and DILI were defined by the treating physician and registered in patient’s medical records. The most common symptoms associated with suspected ADR reported were: pain in the upper abdomen or stomach, unusual tiredness or weakness, diarrhea, dizziness, nausea and vomiting, skin itching or rash, fever, arthralgia, numbness or tingling in the hands or feet, blurred vision. The clinical presentation of DILI predominantly is acute hepatitis and/or cholestasis marked by increased values of alkaline phosphatase and/or alanine aminotransferase and international normalized ratio. We composed one co-morbidity group including patients with Diabetes Mellitus (DM), human immunodeficiency virus (HIV) and malignancy. Age was divided into two groups (≤34 and ≥ 35 years old) and weight into three groups (<50kg, 50–75 kg and >75kg) as defined in the national tuberculosis registry. Race was classified according to geographical affiliation: European / Caucasian background referred to patients who originated from countries above the line of sub-Saharan Africa, African referred to patients originating from countries in sub-Saharan Africa, Asian referred to origin from one of Asian countries.

As this was a retrospective study using routinely collected data, ethical clearance was not required under Dutch Law.

## Analysis

Demographic characteristics of the two patients groups were described and tested for possible differences. Univariable logistic regression was performed for each individual variable of interest. We conducted logistic regression analysis to identify the frequency of each of the measured patient characteristics in each of the two groups, and to identify those associated with extended TB treatment. Variables that showed a significant association (P<0.05) in the univariable analysis and those that a priori were considered to be potential confounders as like suspicious symptoms for ADR and comorbidity were introduced in the multivariable logistic regression model. All analyses were performed in SPSS 22.0 (Chicago, IL, USA).

## Results

**Fig 1:**
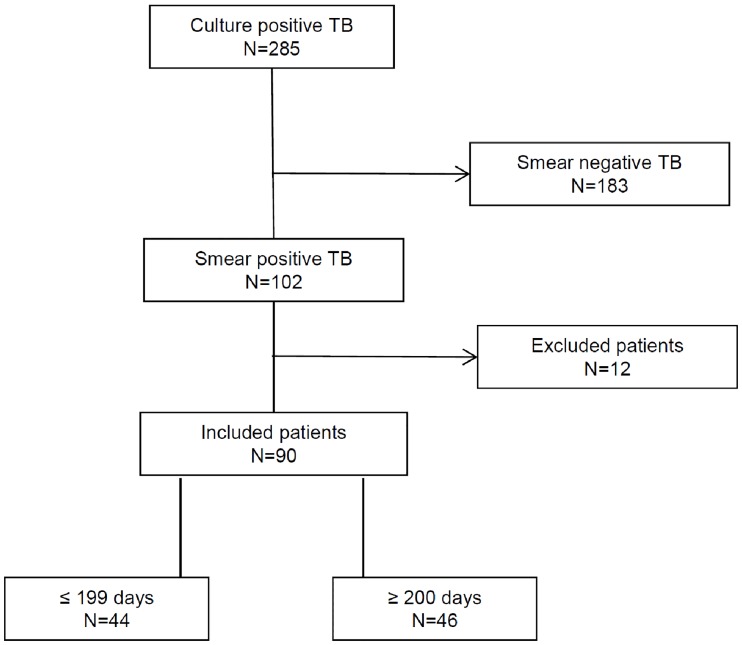
Flow chart showing the participants included in the analysis.

Out of the 285 culture positive TB patients reported between January 2010 and December 2013 in region Haaglanden, 102 (36%) patients had smear- and culture- positive pulmonary TB. In total follow-up data of 8 patients were not available, 2 patients had a final diagnosis of Nontuberculous Mycobacterial Disease and 2 died during the intensive phase of TB treatment. The cause of death of both patients was a combination of old age, underlying comorbidities and fulminant miliary TB. Thus 90 patients out of 102 were eligible for analysis: 46 patients were treated ≥ 200 days and 44 patients were treated ≤ 199 days (Fig 1). In 13 of 46 cases (28%) the extension of treatment occurred in the intensive phase.

Table 1 shows the distribution of demographic, TB diagnosis and TB treatment patient characteristics between two groups and crude ORs assessed for their association with extension of treatment.

**Table 1:** Analysis of patient related characteristic associated with extended treatment.

Variable		Treatment ≥ 200 days (n = 46),n (%)	Treatment ≤ 199 days (n = 44),n (%)	OR	95% CI	P-value
**Demographic characteristics**						
Gender	Female	15 (33)	18 (41)	1.00		
	Male	31 (67)	26 (59)	1.43	0.60–3.40	0.42
Age, y	≤ 34	29 (63)	23 (52)	1.00		
	≥ 35	17 (37)	21 (48)	0.64	0.27–1.50	0.30
Weight (kg)	< 50	3 (7)	2 (5)	1.00		
	50–75	35 (76)	37 (83)	0.59	0.93–3.8	0.58
	> 75	8 (17)	5 (12)	0.93	0.11–7.8	0.95
Race	African	8 (17)	7 (16)	1.00		
	Caucasian	24 (52)	25 (57)	0.84	0.26–2.7	0.77
	Asian	14 (31)	12 (27)	1.02	0.28–3.7	0.96
Addiction	No	34 (74)	32 (73)	1.00		
	Yes	12 (26)	12 (27)	0.94	0.37–2.4	0.90
**TB diagnosis characteristics**						
Disease site	PTB	39 (85)	41 (93)	1.00		
	PTB/EPTB	7 (15)	3 (7)	2.43	0.59–10.2	0.22
X-ray: cavitary TB	No	24 (52)	29 (66)	1.00		
	Yes	22 (48)	15 (34)	1.77	0.74–4.2	0.20
TB treatment history	No	40 (87)	40 (91)	1.00		
	Yes	6 (13)	4 (9)	1.54	0.40–5.9	0.52
Time to smear conversion	<1 month	13 (28)	13 (30)	1.00		
	1–2 month	10 (22)	14 (32)	0.71	0.23–2.2	
	>2 month	11 (24)	1 (2)	11.00	1.20–98	0.96
	missing data	12 (26)	16 (36)	0.75	0.26–2.2	
Time to culture conversion	<1 month	9 (20)	14 (32)	1.00		
	1–2 month	8 (17)	11 (25)	1.33	0.32–3.9	
	>2 month	11 (24)	2 (5)	8.56	1.5–48	0.91
	missing data	18 (39)	17 (38)	1.65	0.57–4.8	
**TB treatment characteristics**						
Creatinine (mmoL/L)	< 50	5 (11)	5 (12)	1.00		
	> 50	41 (89)	39 (88)	1.17	0.27–5.1	0.83
Symptoms of ADR	No	23 (50)	31 (70)	1.00		
	Yes	23 (50)		2.39	1.0–5.7	0.05
DOT	No	27 (59)	31 (70)	1.00		
	Yes	19 (41)	13 (30)	1.69	0.70–4.0	0.25
Co-morbidity	No	36 (78)	38 (86)	1.00		
	Yes	10 (22)	6 (14)	1.8	0.58–5.3	0.13
DILI	No	35 (76)	43 (98)	1.00		
	Yes	11 (23)	1 (2)	13.51	1.7–109.8	0.02
TB sanatorium hospitalization	No	42 (91)	38 (86)	1.00		
	Yes	4 (9)	6 (14)	0.60	0.16–2,3	0.46
Treatment by specialist of:	GGD	7 (15)	10 (23)	1.00		
	Hospital	39 (85)	34 (77)	1.64	0.56–4.8	0.37

OR, odds ratio; CI, confidence interval; PTB, Pulmonary tuberculosis; EPTB, Extrapulmonary Tuberculosis; Co-morbidity: HIV, DM and malignancy; ADR: adverse drug reaction; DOT: Direct Observed Treatment; GGD: TB Municipal Health Service.

The study groups did not differ in terms of ethnicity, gender and age. The median age of patients in both groups was similar, 32 versus 34 years. Caucasians prevailed in both groups.

None of the demographic characteristics of patient groups were statistically significantly associated with extended treatment duration. Despite the high proportion of patients with DM treated ≥ 200 days, 8 versus 4 patients, co-morbidity also was not significantly associated with extension of treatment (OR = 1,8; 95% CI 0.58–5.3). Besides, the group of patients treated ≥ 200 days included a higher proportion of patients with a combination of extra-pulmonary and pulmonary TB; with cavitary lesions; and a history of previous TB treatment. These factors were not significantly associated either, although they demonstrated moderate positive association. Out of the 10 patients reported with EPTB 7 were from group of prolonged treatment: 2 with intrathoracic lymph node TB, 1 with laryngitis, 2 with peripheral lymph node TB and 2 patients had a TB meningitis. Three patients from standard group had intrathoracic lymph node TB, pleural and cervical spinal TB respectively. The optimal duration of treatment of TB meningitis caused by susceptible strains is not defined. There are no clinical trials comparing a six-month to a longer treatment regimen for TB meningitis. Six- to nine-month regimes containing R for treatment of osteoarticular TB are at least as effective as 18-month regimens without R (1). Both included patients with TB meningitis had complex interrelations between TB and comorbidity caused the extension of their treatment.

Typical finding in our study was the lack of microbiological tests. This can be explained by the absence of sputum production and the reluctance of clinicians to repeat bronchoalveolar lavage. In total 36 (40%) patients, 21 with extended treatment and 15 with standard treatment had a cavitation on the initial chest X-ray and just 6 (17%) of them, 5 from the group of extended treatment duration, and 1 from standard treatment duration, were still sputum smear positive at the end of the intensive phase. Presumed ADR was associated with extended treatment (OR 2.39, 95% CI: 1.01–5.7). The development of DILI was strongly associated with extended treatment duration (OR: 13.5; 95% CI: 1.7–109.8). Results of time of smear and culture conversion were available for 31% and 39% of patients respectively. For those with results available, univariable regression analyses showed a significant association between longer than 2 month positive smear (OR: 11.00; 95% CI: 1.24–98) and culture conversion rate (OR: 8.56; 95% CI: 1.53–48) with extension of the total TB treatment duration. The results of the multivariable logistic regression analysis are presented in Table 2.

**Table 2:** Multivariable analysis: conditional logistic regression model for predictors of prolonged TB treatment.

		Unadjusted OR		Adjusted OR	
Variable and category		OR (0.95%CI)	P- value	OR (0.95%CI)	P- value
**Time Smear Conversion**	<1 month	1.00		1.00	
	1–2 month	0.71 (0.23–2.2)		0.33 (0.67–1.6)	
	>2 month	11.00 (1.2–98.0)	0.96	2.20 (0.16–30.8)	0.19
	missing data	0.75 (0.26–2.2)		0.29 (0.05–1.5)	
**Time Culture Conversion**	<1 month	1.00		1.00	
	1–2 month	1.33 (0.32–3.9)		1.43 (0.23–8.7)	
	>2 month	8.56 (1.5–48.0)	0.91	6.02 (0.58–62.1)	0.33
	missing data	1.65 (0.57–4.8)		12.98 (0.56–15.8)	
**DILI**	No	1.00		1.00	
	Yes	13.51 (1.7–109.8)	0.02	13.62 (1.47–126.6)	0.02
**Symptoms of ADR**	No	1.00		1.00	
	Yes	2.39 (1.0–5.7)	0.05	1.72 (0.59–5.0)	0.32
**Co-morbidity**	No	1.00		1.00	
	Yes	1.8 (0.58–5.3)	0.13	0.94 (0.23–3.8)	0.93

If we adjusted for the potential confounders time to culture conversion and co-morbidity, DILI remained associated with extension of treatment duration (OR = 13.62, 95% CI 1.47–126.6), but this association with symptoms indicative for ADR disappeared (OR = 1.72, 95% CI 0.59–5).

## Discussion

To the best of our knowledge this is the first study to analyze predictors for prolonged TB treatment in a Dutch outpatient setting. Univariable analysis showed a statistically significant association between ‘symptoms’, DILI and smear and culture conversion more than two months after start of treatment with extended TB treatment duration. In multivariable analysis just one single factor- the development of DILI during the TB treatment—contributed significantly to the extension of TB treatment. It needs to be mentioned that we could only identify 11 patients with DILI and extended treatment that could be compared with 1 DILI patient without extended treatment time. This resulted in wide confidence intervals in logistic regression analysis. A larger study could be helpful to confirm this finding.

Nearly one in four patients (23%) from the group of patients treated ≥ 200 days in our study was registered with DILI during TB treatment, compared to 2% in the standard treatment group. This is in line with earlier publications that showed the risk of DILI in diverse studies to range from 5 to 33% [20–22]. The pathogenesis and types of DILI vary from hepatic adaptation to hepatocellular injury. Knowledge of the mechanism of TB DILI is still incomplete. The number of different studies on factors contributing to development of hepatotoxicity during the TB treatment indicated age, ethnicity, pregnancy, malnutrition, alcohol consumption, HIV and viral hepatitis as predictors of TB DILI [18,23,24].

In case of elevation of liver enzymes during the TB treatment, a serum level more than three times the upper limit of normal in the presence of symptoms, or more than five times the upper limit of normal in the absence of symptoms, an interruption of treatment is recommended by different guidelines [1,17,25]. The variation of outcomes of DILI, ranging from spontaneous resolution to liver failure and death, can sometimes lead to extreme caution resulting in early interruption of treatment by clinicians. Reintroduction of treatment is possible if the level of liver enzymes has decreased to less than two times the upper limit of normal and symptoms have significantly improved.

Multifaceted social issues are reported associated with adherence to TB treatment and treatment duration. Most of these social-related factors are country-specific. Therefore TB is known as the barometer of social welfare. Homeless, financial insecurity, lack of effective social support, criminality, and addiction problems make these patients more vulnerable and the treatment of TB more complicated [26–29]. The Netherlands as like most low-incidence countries has a relatively well-financed health system with favourable conditions for treatment of TB [30]. The identification of those factors with social character, associated with unfavorable outcomes, at the beginning of treatment in order to implementation of DOT is very important. In the Netherlands all TB patients are supervised and supported during treatment by the public health nurse of the MPHS, using diverse case management interventions. Since 2007 the DOT coverage has reached nearly 80% among unemployed and substance abusers, and 90% among the prison population. During the study period 2012–2013 the percentage of TB-patients in risk groups decreased further resulting in a stop of the periodic screening of drug addicts and homeless in Haaglanden region due to the low yield and therefore less effect of the screening [31].

The WHO guideline does not suggest measurement of drug concentration in selected clinical cases like these and reintroduction schedules do not consider drug concentration measurements. It seems reasonable to measure the blood concentration of anti TB drugs in patients diagnosed with hepatic dysfunction, to be able to adapt the dose tailored to the individual patient. In this perspective the results of a recently published study from India are very remarkable. Satyaraddi et al. reported higher plasma level of Rifampicin (R) in 15 TB patients who developed DILI in comparing with 95 patients who did not [32]. The maximum concentration of R with cut-off 12.50 mg/L predicted subsequent development of DILI in 93.3% of the patient, which may prevent the occurrence of DILI.

There are a number of ongoing efforts in different fields of pathophysiology of this clinical complication. Studying the effect of genetic variation is one of them. Results of prospective randomized controlled study of Azuma and colleagues showed a great potential of the genotype-guided dosing stratification of Isoniazid (INH) in chemotherapy of TB and development of liver injury [33]. These conclusions are concordant with results of studies conducted by Sotsuka et al. and Wang et al. reporting drug-metabolizing enzyme polymorphisms and predisposition to DILI [34,35]. Moreover, several studies evaluated the relation between drug concentrations, drug metabolizing enzymes and toxicity. Although findings were consistent, showing toxicity at higher concentrations, a randomized controlled trial showing that TDM reduces DILI has not been performed [36–38].

In our study we have studied culture conversion rates as potential indicator of extended TB treatment, related to insufficient drug exposure. According to the WHO the extension of the TB therapy is recommended if sputum-culture examination at the end of the second month of treatment is still positive for patients who have cavitation on the initial chest radiograph. The presence of cavitary disease in our study did not have a significant association with extended treatment but this could have been caused by the small sample size. Twenty-four percent of the patients who received extended treatment had delay of sputum smear and culture conversion longer than 2 months. This outcome well corresponded with results of several epidemiological studies indicating that the proportion of TB patients who remain smear-positive after 2 months of treatment can be greater than 20% (3.3–25.3%) [39–48]. However, our multivariable regression analysis did not confirm significant differences in delay of culture conversion between our two groups. Data linking insufficient drug levels to delay of culture conversion is scarce. A retrospective cohort study of Heysell et al. conducted in Virginia, identified lower than expected levels of INH, R and Ethambutol (E) among patients with delayed treatment response [49]. Results of a prospective study of Pasipanodya et al. conducted in Western Cape, South Africa confirmed also the predictive role of low drug exposure in poor long-term clinical outcomes in 25% of TB patients [50]. As our study show no difference in culture conversion rate it’s unlikely that low drug exposure could have been the underlying cause of prolonged treatment. At this moment there is a growing understanding of the link between the plasma concentration of anti TB drugs and efficacy and adverse events and its potential role in the management of TB treatment [51,52]. As a result, in 2014, TBNET published a consensus statement of management of drug-resistant TB in Europe, advising TDM with drug dose adjustment [11]. The Recently published clinical practice Guideline of ATS/CDC/IDSA for treatment of drug-susceptible TB, endorsed by European Respiratory Society, for the first time provides recommendations in which conditions or situations TDM might be helpful. Patients with a medical conditions suspected of leading to subtherapeutic or toxic drug concentrations is one of the examples [53].

The present study has several limitations that should be mentioned. An important limitation was the relatively small cohort study. The Netherlands is a low-burden TB country with incidence of tuberculosis 6,5 in 2010 decreased till 5,1 in 2013 with just around 20–23% patients with smear and culture positive pulmonary drug-sensitive TB. Moreover, TB in western European is concentrated in urban areas, specifically in the Netherlands in 4 big cities: The Hague, Rotterdam, Amsterdam and Utrecht, without significant difference in patient’s population [54–56]. Our findings based on data of TB patients from region Haaglanden would be not essentially different between the regions and therefore reflect the situation in the Netherlands, at least in period between 2010–2013. Due to the retrospective nature of the study patients ‘records were partly incomplete. The main reason was that part of the treatment was performed in a hospital or other public health region. Final results of these cases were reported in the NTR. Due to incomplete data we excluded these cases. Although already known before start of the study, actual drug concentrations and values of minimal inhibitory concentrations were not available as these were not part of standard treatment. Lack of both microbiological tests can be partly explained, on the one hand, by the absence of sputum production, and on the other hand by the reluctance of clinicians to repeat bronchoscopic bronchoalveolar lavage, an invasive and patient unfriendly procedure. Despite these limitations the results of this study showing DILI as an independent risk factor for extended treatment justify a prospective study to detect whether pharmacokinetics and pharmacogenetics are associated with DILI. A better understanding of factors associated with delayed treatment response and ADR, may help to improve management of patients receiving TB treatment.

## Conclusion

DILI was identified as a significant independent predictor of extended TB treatment. This study justifies a prospective investigation evaluating whether potential factors like pharmacokinetics and pharmacogenetics are associated with DILI. A better understanding of factors associated with DILI and delayed treatment response, may help to improve management of patients receiving TB treatment.
